# Assessing the Quality of Decision Support Technologies Using the International Patient Decision Aid Standards instrument (IPDASi)

**DOI:** 10.1371/journal.pone.0004705

**Published:** 2009-03-04

**Authors:** Glyn Elwyn, Annette M. O'Connor, Carol Bennett, Robert G. Newcombe, Mary Politi, Marie-Anne Durand, Elizabeth Drake, Natalie Joseph-Williams, Sara Khangura, Anton Saarimaki, Stephanie Sivell, Mareike Stiel, Steven J. Bernstein, Nananda Col, Angela Coulter, Karen Eden, Martin Härter, Margaret Holmes Rovner, Nora Moumjid, Dawn Stacey, Richard Thomson, Tim Whelan, Trudy van der Weijden, Adrian Edwards

**Affiliations:** 1 Department of Primary Care and Public Health, School of Medicine and the School of Psychology, Cardiff University, Cardiff, United Kingdom; 2 Ottawa Health Research Institute, University of Ottawa, Ottawa, Ontario, Canada; 3 School of Nursing, University of Ottawa, Ottawa, Ontario, Canada; 4 W. Alpert Medical School, Brown University, Centers for Behavioural and Preventive Medicine, Providence, Rhode Island; 5 Department of Internal Medicine, University of Michigan, Ann Arbor, Michigan, United States of America; 6 Maine Medical Center, Center for Outcomes Research and Evaluation, Portland, Maine, United States of America; 7 Picker Institute Europe, King's Mead House, Oxford, United Kingdom; 8 John M. Eisenberg Clinical Decisions and Communications Science Center, Department of Medical Informatics and Clinical Epidemiology, Oregon Health&Science University, Portland, Oregon, United States of America; 9 Institute and Policlinic for Medical Psychology, University Medical Center Hamburg-Eppendorf, Hamburg, Germany; 10 Center for Ethics, College of Human Medicine, Michigan State University, East Lansing, Michigan, United States of America; 11 Centre Léon Bérard, University of Lyon, Lyon, France; 12 Institute of Health and Society, Medical School, Framlington Place, University of Newcastle, Newcastle upon Tyne, United Kingdom; 13 Department of Oncology, McMaster University, Juravinski Cancer Centre, Hamilton Ontario, Canada; 14 Department General Practice, School for Public Health and Primary Care (CAPHRI), Maastricht University, Maastricht, the Netherlands; University of California San Francisco, United States of America

## Abstract

**Objectives:**

To describe the development, validation and inter-rater reliability of an instrument to measure the quality of patient decision support technologies (decision aids).

**Design:**

Scale development study, involving construct, item and scale development, validation and reliability testing.

**Setting:**

There has been increasing use of decision support technologies – adjuncts to the discussions clinicians have with patients about difficult decisions. A global interest in developing these interventions exists among both for-profit and not-for-profit organisations. It is therefore essential to have internationally accepted standards to assess the quality of their development, process, content, potential bias and method of field testing and evaluation.

**Methods:**

Scale development study, involving construct, item and scale development, validation and reliability testing.

**Participants:**

Twenty-five researcher-members of the International Patient Decision Aid Standards Collaboration worked together to develop the instrument (IPDASi). In the fourth Stage (reliability study), eight raters assessed thirty randomly selected decision support technologies.

**Results:**

IPDASi measures quality in 10 dimensions, using 47 items, and provides an overall quality score (scaled from 0 to 100) for each intervention. Overall IPDASi scores ranged from 33 to 82 across the decision support technologies sampled (n = 30), enabling discrimination. The inter-rater intraclass correlation for the overall quality score was 0.80. Correlations of dimension scores with the overall score were all positive (0.31 to 0.68). Cronbach's alpha values for the 8 raters ranged from 0.72 to 0.93. Cronbach's alphas based on the dimension means ranged from 0.50 to 0.81, indicating that the dimensions, although well correlated, measure different aspects of decision support technology quality. A short version (19 items) was also developed that had very similar mean scores to IPDASi and high correlation between short score and overall score 0.87 (CI 0.79 to 0.92).

**Conclusions:**

This work demonstrates that IPDASi has the ability to assess the quality of decision support technologies. The existing IPDASi provides an assessment of the quality of a DST's components and will be used as a tool to provide formative advice to DSTs developers and summative assessments for those who want to compare their tools against an existing benchmark.

## Introduction

There has been increasing interest in the use of ‘decision aids’ [1], defined as adjuncts to the discussions clinicians have with patients during deliberations about decisions: these aids provide information about options and help clarify personal values [2]. These adjuncts range from leaflets through face to face methods such as coaching or counselling to interactive multimedia websites. To describe this generic family of clinician-patient interventions we will use the term *decision support technologies* (DSTs) [3], corresponding with the internationally recognised need to assess the impact of ‘health technologies’ [4]. DSTs are complex interventions which require detailed assessment to ensure safe use in healthcare contexts [3] because they help make options explicit, provide information about harms and benefits, clarify patient values' and provide structured means to help people deliberate when making decisions. Although there are published methods to assess the quality of clinical practice guidelines [5], DSTs go further and address issues of equipoise for which patients need to deliberate about difficult choices [6]. However, as yet, there are no reliable methods to assure the quality of DSTs development process, content, potential bias, and method of field testing and evaluation – a gap which we address in this study. We did not intend to develop methods to assess how DSTs are used in practice, in the clinical encounter, although we recognise that this is an important area that requires further work.

There are reports that DSTs have achieved a ‘tipping point’ in the US and are widely accessed by increasing numbers of patients [1]. The ability of DSTs to improve the quality of decisions and enable reductions in discretionary surgery and invasive procedures without adverse effects on health outcomes has been demonstrated in clinical trials [2], [7]. The central role that these technologies will play in future healthcare systems is increasingly recognised [1], [8]–[10]. Over the last decade, the interest in developing DSTs has moved beyond research groups and has entered the commercial world. A global interest in developing DSTs has emerged among both for-profit and not-for-profit organisations. It is therefore essential to have a set of internationally accepted standards to assess their quality, to assess whether interests are declared and whether they are unduly biased [8], [9].

The International Patient Decision Aid Standards (IPDAS) Collaboration produced a checklist for the assessment of DSTs [11]. The checklist was rigorously developed in a two stage web-based Delphi process using online rating process to enable international collaboration. A total of 122 individuals from four stakeholder groups (researchers, practitioners, patients, policy makers) representing 14 countries reviewed background evidence summaries, and rated the importance of 80 criteria in 12 quality dimensions. Second round participants received feedback from the first round and repeated their assessment of the 80 criteria plus three new ones. The IPDAS checklist enabled broad assessments in 12 dimensions: systematic development process, providing information about options; presentation of probabilities; clarification of values; use of patient stories; information about guiding or coaching; disclosure of interests; providing internet access; balanced presentation of options; use of plain language; use of up-to-date evidence; and effectiveness. The IPDAS checklist allows users, developers and others to assess whether these technologies contain the suggested components and judge whether they underwent rigorous development and evaluation. It has been used in updating the Cochrane systematic review of DSTs and to guide the development of DSTs [12], [13].

However, the checklist was not designed to provide precise, quantitative assessments, such that judgements could be made about the quality of DSTs, either at item, dimension or global levels. In addition, because not all checklist items were applicable to every DST, comparability, even at the checklist level, was not possible. Given interest in being able to assess these DSTs at a more precise level of detail — in terms of how they were developed and field tested, whether their content was valid and whether effectiveness had been evaluated with patients facing relevant decisions — the IPDAS Collaboration agreed that achieving this objective would require an instrument capable of quantitatively assessing the quality of DSTs. The aim of this article is to describe the development, validation and inter-rater reliability of an IPDAS instrument (IPDASi), built on the existing framework.

## Methods

IPDASi was developed in four stages.

### Stage 1 Refinement and preparation of instrument (IPDASi v1)

The published IPDAS checklist required transformation into a quantitative instrument, although we agreed to adopt the dimension-item framework. As part of this preparation, a group of researchers (GE, DS, RT, CB, SB, TW) used the existing checklist and dimension-item framework to score three purposefully selected DSTs, representing different design approaches and where our prior overall assessments indicated variable quality. These were Healthwise's Breast Cancer Surgery (BCS), web-based information, Bastian&McBrides Hormone Replacement Therapy (HRT), an illustrated booklet, and Wolf et al's Prostate Specific Antigen (PSA) screening, a brief text-based script. A binary (yes/no) and ‘not applicable’ scale was proposed; comments were collected on item applicability. Tabulations and qualitative analyses were performed but inter-rater correlations were not calculated.

### Stage 2 IPDASi Confirmation of items (IPDASi v2)

On the basis of the results of Stage 1, a refined version IPDAS instrument (IPDASi v2) was designed and used in Stage 2. The non-applicable option was removed, and in this and all subsequent versions, a 4-point rating scale was used for each item, with possible responses as follows: strongly agree = score 4 (the issue is addressed clearly and comprehensively); agree = score 3 (the issue is addressed but with room for improvement); disagree = score 2 (the DST fails to clearly address the issue); strongly disagree = score 1 (the DST totally fails to address the issue). In common with the binary (yes/no) scale it replaced, the scale intentionally does not include a midpoint expressing neutrality. Items in the ‘balance’ dimension were integrated into the ‘information’ dimension. The web dimension was not applicable to all DSTs, therefore removed. A website was created for data collection (http://www.ipdasi.org/). Scale anchor point descriptions were developed for all items.

Five raters, two in the UK (MA-D and SS, Cardiff) and three in North America (ED and SK in Ottawa and MP in Providence) were familiarised with IPDASi v2, prior to using it to score the three previously selected DSTs, and asked to comment on item phrasing. Members of the IPDASi development group were asked to view the IPDASi instrument online and comment on item phrasing. For IPDASi v2 and subsequent versions, item scores were rescaled to be 0 to 100. At Stage 2, only an unweighted average of all items was calculated, as our focus was not on dimension scores. Analysis included inter-rater reliability using intraclass correlations for two way random effects at item and global score levels [14].

### Stage 3 IPDASi Validation Study

Based on the results of Stage 2, a third version, IPDASi v3 was designed. This retained the majority of items from Stage 2, albeit with changes to phrasing. It comprised 47 items representing 10 dimensions. 9 dimensions applicable to all DSTs relate to Information (8 items); Probabilities (8 items); Values (4 items); Decision Guidance (2 items); Development (6 items); Evidence (5 items); Disclosure (2 items); Plain language (1 item); Evaluation (2 items). One additional dimension (9 items) relates to decisions based around tests or screening. Feedback from the comments resulted in more detailed anchor scale descriptions and standardization of descriptions.

IPDASi v3 was then used in a validation study to assess the quality of a sample of DSTs. Two approaches were used to achieve a sample of DSTs. First, five major producers of publically available DSTs were identified (The Foundation for Informed Medical Decision Making, Healthwise, Mayo Clinic, Midwives Information and Resource Service (MIDIRS) and Ottawa Health Decision Centre (OHDeC). Three DSTs from each producer were chosen at random, giving a total of 15. Second, 66 English-language DSTs, for which contact details were available, were chosen at random from the Cochrane inventory maintained by the University of Ottawa (http://decisionaid.ohri.ca/cochinvent.php), and their developers were approached and asked:

1. Whether the DST was in current use and free of charge to clients;
2. For consent to assess the DST using IPDASi; and
3. For copies or information about documentation (published reports or peer reviewed articles) about the development or evaluation of the DST.

Each DST included in the sample was prepared for assessment in a standardised way. Background documents (relevant publications, reports) and all DST content were made available online (either in pdf or html formats; videos were converted into Windows Media Video format) for raters to assess. Table 1 provides details of the DSTs that were included in the sample, and the results of the IPDASi assessments.

**Table 1:** Thirty sampled decision support technologies: sample characteristics and adjusted full IPDASi (v3) * and SF scores based on duplicate assessment, with 95% confidence limits.

ID#	Raters	Developer	Title	Topic Area	Format	Length # Pages/Duration	IPDASi Scores	
							Adjusted Weighted (Lower and Upper limit)	SF (Lower and Upper limit)
32	NJ, ED	Wakefield, MU	Genetic testing for breast cancer ovarian cancer risk: A decision aid for people with a family history of breast and/or ovarian cancer	Breast and ovarian cancer screening	Text (paper)	v1 40 pp, v2 32 pp	81.5 (74.3–88.8)	83.1 (72.1–94.2)
1050	NJ, MP	OHDeC	Should you have a steroid injection for tennis elbow?	Tennis elbow treatment	Text (paper, PDF)	7 pp	77.4 (70.2–84.6)	72.5 (61.5–83.4)
1197	MS, ED	OHDeC	Should you take steroids and immunosuppressive agents for lupus kidney disease?	Lupus kidney disease treatment	Text (paper, PDF)	7 pp	71.9 (64.7–79.1)	72.4 (61.4–83.4)
1174	SS, SK	OHDeC	Long Term Feeding Tube Placement in Elderly Patients	End of life treatment	Web site or Text (paper, PDF)	37 Web pp with audio 23 pages	69.2 (62.1–76.4)	67.4 (56.5–78.2)
35	MAD, AS	MCC	Making the Choice: What to do about early stage prostate cancer	Prostate cancer treatment	Web site or Text (paper, PDF)	49 Web pp	65.5 (58.3–72.7)	80.9 (69.9–91.9)
						28 pp		
48	MS, SK	Wakefield, MU	Genetic testing for hereditary non-polyposis colorectal cancer (HNPCC): A decision aid for people with a family history of HNPCC	Colorectal cancer: genetic testing	Text (paper)	40 pp	65.1 (57.8–72.3)	67 (56–78)
3	MAD, AS	Shorten, ACM	Birth Choices	Vaginal birth after caesarean	Text (paper)	14 pp	64.0 (56.8–71.2)	73.6 (62.6–84.6)
17	MS, MP	Elwyn, CU	Prosdex	Prostate cancer screening	Web site	84 Web pp with audio&12 video clips	62.5 (55.2–69.8)	68.2 (57.1–79.4)
37	MAD, SK	Col, CORE	Women's Interactive System for Decisions on Menopause	Menopause treatment	Web site (portions of Web site unavailable at time of review)	441 Web pp	62.0 (54.9–69.2)	59.2 (48.4–70.1)
13	MAD, ED	Leighl, UoT	Decision Aid for Patients with Metastatic Colorectal Cancer Facing a Treatment Decision	Colorectal cancer treatment	Text (paper)	32 pp	62.0 (54.9–69.2)	74.7 (63.8–85.6)
1046	MAD, SK	Mayo Clinic	Birth Control Guide	Family planning	Web site	63 pp	61.1 (54.0–68.3)	59.2 (48.4–70.1)
1023	SS, MP	FIMDM	Treatment Choices for Coronary Artery Disease	Coronary artery disease treatment	Video	51 min	56.1 (48.9–63.3)	61.6 (50.6–72.6)
54	SS, MP	APCC	Localized Prostate Cancer: A guide for men and their families	Prostate cancer treatment	Text (paper)	103 pp	54.8 (47.6–62.0)	53.2 (42.2–64.2)
53	SS, AS	Lawrence, STVHCS	Mammography Decision Aid	Breast cancer screening	Decision board&script	4 pp	52.6 (45.3–59.8)	63.2 (52.2–74.2)
						7 pp		
1012	MS, MP	Healthwise	Should I take medicine for high blood pressure?	High blood pressure treatment	Web site	18 pp	51.8 (44.4–59.1)	35.5 (24.4–46.6)
1121	NJ, SK	FIMDM	Hormone Therapy: When the PSA rises after prostate cancer treatment	Prostate cancer treatment	Video&Text (paper)	37 min	51.7 (44.5–59.0)	57.4 (46.4–68.4)
						29 pp		
1090	MS, AS	FIMDM	Colon Cancer Screening: Deciding What's Right For You	Colon cancer screening	Video&Text (paper)	32 min	50.5 (43.3–57.7)	56.9 (46–67.9)
						21 pp		
1011	MAD, ED	Healthwise	Should I take antibiotics for acute bronchitis?	Acute bronchitis treatment	Web site	33 pp	49.0 (41.8–56.1)	41.4 (30.5–52.2)
15	SS, SK	Barratt, UoS	Should I Start Having Mammograms to Screen for Breast Cancer?	Breast cancer Screening	Web site	15 Web pp	48.4 (41.3–55.6)	55.8 (44.9–66.7)
64	MAD, MP	Taylor, GU	The Right Decision is Yours: A Guide to Prostate Cancer Check-Ups	Prostate cancer screening	Text (paper)	19 pp	46.1 (38.9–53.4)	44.7 (33.7–55.7)
1059	MAD, MP	MIDIRS	If your baby is in the breech position, what are your choices?	Breech birth	Text (paper)	13 pp of 122 pp booklet	45.1 (37.8–52.3)	39.9 (28.9–50.9)
1150	SS, AS	Mayo Clinic	Enlarged prostate (BPH) guide	Benign prostatic hypertrophy treatment	Web site	69 Web pp with 11 video clips	44.7 (37.5–52.0)	46.3 (35.3–57.3)
1067	NJ, AS	Healthwise	Should I have tests for irritable bowel syndrome?	Irritable bowel syndrome screening	Web site	31 pp	44.1 (36.8–51.4)	34.5 (23.3–45.6)
49	MS, ED	Crouch, Baylor	Statin Therapy Informed Choice	High cholesterol treatment	Text (paper)	9 pp	43.9 (36.7–51.1)	53.6 (42.6–64.6)
1155	NJ, ED	MIDIRS	Ultrasound scans: what you need to know	Prenatal screening	Text (paper)	13 pp of 122 pp booklet	43.5 (36.2–50.7)	39.3 (28.3–50.3)
6	SS, ED	NERI	Urinary Incontinence: Finding the Solution	Urinary incontinence	Video: Male	27 min	43.3 (36.1–50.4)	48.5 (37.6–59.3)
					Female	21 min		
12	NJ, SK	NERI	Making the Right Choice: Decision aid for prostate cancer	Prostate cancer treatment	Video	39 min	43.2 (35.9–50.4)	53.3 (42.3–64.3)
1061	SS, ED	Mayo Clinic	Carpal tunnel syndrome guide	Carpal tunnel treatment	Web site	50 Web pp with 14 video clips	39.3 (32.2–46.5)	40.1 (29.2–51)
1056	MS, SK	MIDIRS	Place of birth	Location of child birth	Text (paper)	11 pp of 122 pp booklet	37.3 (30.0–44.5)	36.1 (25.1–47.1)
1	NJ, AS	US CDC	Prostate Cancer Screening. A decision guide for African Americans	Prostate cancer screening	Web site or Text (paper, PDF)	1 p	32.9 (25.6–40.3)	44.1 (33–55.2)
						20 pp		

* Adjusted scores: scores from the two raters were adjusted to take account of their personal propensity to give higher or lower scores. Components of variation were modelled by Bayesian modelling (Markov chain Monte Carlo) using WinBugs software, leading to estimated confidence intervals.

**Abbreviations**: (APCC: Australian Prostate Cancer Collaboration; Barratt, UoS: University of Sydney; Crouch, Baylor: Baylor College of Medicine; Col, CORE: Center for Outcomes Research and Evaluation; Elwyn, CU: Cardiff University; FIMDM: Foundation for Informed Medical Decision Making; Lawrence, STVHCS: South Texas Veterans Health Care System; Leighl, UoT: University of Toronto; MCC: Michigan Cancer Consortium Prostate Cancer Action Committee; MIDIRS: Midwife Information and Resource Service; NERI: New England Research Institutes; OHDeC: Ottawa Health Decision Centre; Shorten, ACM: Australian College of Midwives; Taylor, GU: Georgetown University; US CDC: Centers for Disease Control and Prevention; Wakefield MU, Macquarie University).

Eight raters with diverse backgrounds and training were trained to undertake independent ratings: four in the UK (MA-D, MS, NJ, SS in Cardiff) and four in North America (SK, ED, AS in Ottawa; MP in Providence). Each DST was scored by two raters, one chosen randomly from each location, such that one rating was done in UK and the other in North America. New raters were asked to pilot the instrument on a ‘test’ DST and new raters also had access to raters who had completed the Stage 2 assessment if they required advice on item interpretation.

As in Stage 2, each item was scored on a 4-point scale, rescaled from 0 to 100, and dimension means were calculated. Two overall scores were calculated, scaled 0 to 100: the unweighted mean of all items (38 or 47, depending on whether the DST addressed a treatment or a test/screening decision) and the weighted mean score, a mean of the 9 or 10 dimension-specific means. The latter score upweights items belonging to dimensions comprising few items and downweights items from dimensions with many, but each dimension contributes an equal weight into the final score.

Summary statistics were calculated for dimension scores and unweighted and weighted overall means. Weighted means were modelled by rater and tool in a two-way balanced incomplete ANOVA model. Intraclass correlations and Cronbach's alpha, by each rater and by dimension means, were also calculated. The quality of each DST was then characterised by the average of the weighted mean scores from the two raters, adjusted by the model to take account of their personal propensity to give higher or lower scores. We wanted to predict the degree of accuracy if others used IPDASi in the future, considering one or two raters, known to us (i.e. one of the existing eight raters) or unknown to us. To achieve this, components of variation were determined by Bayesian modelling (Markov chain Monte Carlo) using WinBugs software [15], to arrive at estimated confidence interval half-widths for differing future rating situations. The raters' qualitative comments were summarised.

### Stage 4 Agreement on IPDASi-SF (short form)

A core set of items was also chosen to develop a ‘short form’ (IPDASi-SF) aiming to test whether a ‘minimum’ quality threshold could be established. By agreement in the development group, these criteria were chosen based on having an *equimedian* score of 9 (i.e. maximum agreement) in the IPDAS consensus process [11]. The equimedian is designed to represent the cumulative distribution function for a population with equal numbers in each of the four stakeholder groups [11]. In addition, core-set items represented key concepts for each dimension. The 19 items selected for the IPDASi-SF consisted of 3 items for tests/screening and 16 others for all DSTs including: Information (4 items: options available, positive features, negative features, and fair comparison); Probabilities (3 items: reference class, event rates, compare probabilities); Values (1 item: personal importance); Development (3 items: patients' needs, impartial review, tested with patients) ; Disclosure (1 item: information about funding); Evaluation (2 items: knowledge, improved decision quality); Evidence (2 items: citations to studies, production date). The three items selected for the test/screening dimension included: next steps, chances of detection, non-symptomatic. These SF items were not highlighted for special attention during the rating process. Unweighted mean scores were calculated (i.e. all SF items and not the means related to their respective dimensions), and correlations (Pearson) with the IPDASi overall mean adjusted weighted score (Table 2).

**Table 2:** Development of IPDASi versions and IPDASi-SF: item retention and dimension merging.

Stage	1	2	3	4
**IPDASi version**	IPDASi v1	IPDASi v2	IPDASi v3	IPDASi SF
**Number of items**	62*	48	47	19
**Assessors/Raters**	Expert group (GE, DS, RT, CB, SB, TW).	Cardiff: MA-D, MS, NJ, SS; North America: SK, ED, AS MP.	Cardiff: MA-D, MS, NJ, SS; North America: SK, ED, AS MP.	Cardiff: MA-D, MS, NJ, SS; North America: SK, ED, AS MP.
**Number of DSTs evaluated**	3	3	30	30
**Dimensions**				
Information	8	8	8	4
Probabilities	10	8	8	3
Values	3	5	4	1
Decision Guidance	3	2	2	–
Development	7	6	6	3
Evidence	6	5	5	2
Disclosure	2	2	2	1
Plain Language	3	1	1	–
Evaluation	7	2	2	2
Test	5	9	9	3
Web-based	6	Items did not meet inclusion assumption of being applicable to all DSTs and were therefore not included.		
Balance	2	This dimension was merged with probabilities.		

* IPDASi v1 is equivalent to the IPDAS Checklist.

¶ Test dimension items applicable to relevant DSTs.

## Results

Table 2 provides a synopsis of the different versions, detailed in the four stages.

### Stage 1 Refinement and preparation of instrument (IPDAS v1)

Results of the seven raters were compared. The number of comments made at the interpretation level and the wide variation in scoring indicated a need for further item development. In addition some items had double criteria. In October 2006, five researchers met (AC, AOC, DS, CB&GE) and, using the results of this Stage, judged each item against two criteria, clarity and feasibility of measurement. All item phrasings were modified and it was decided to base the development of IPDASi on the following assumptions.

1. All items should be *applicable* to the assessment of all DSTs. This enables the computation of a standard quality score per DST with no adjustment for specific content. An exception was made for DSTs designed to guide deliberations about undertaking diagnostic or screening tests. This type of DST would be subject to an additional dimension of items relating specifically to information on test characteristics.
2. All items should meet the criterion of measurement feasibility. At this Stage, we decided to have 10 dimensions in IPDASi, mirroring the dimensions agreed in the IPDAS consensus process. Further information on dimension and items is presented in Stage 3.

### Stage 2 Refinement and preparation of instrument (IPDAS v2)

Mean scores on a 0–100 scale for the three DSTs were as follows, with SDs reflecting inter-rater variation: HRT 68.7 (6.9); BCS 46.0 (6.5); PSA 38.5 (6.4). The intraclass correlation coefficient was 0.89. These results provided sufficient confidence to refine the instrument for a larger reliability study (Stage 3). Qualitative comments revealed where more specific item anchors descriptors were required, achieved collaboratively using a shared online spreadsheet. Discussions regarding dimension weighting led to agreement that the mean of each dimension should contribute equally to the total score.

### Stage 3 Dual rater assessments of 30 DSTs (IPDAS v3)

Table 1 describes the sample of DSTs and provides the results. Table 3 lists the items used in IPDAS v3. Three DSTs were assessed from each of the five selected major producers. The other 15 were obtained by approaching 36 developers (representing 47 DSTs). Eighteen developers did not respond and we found that five of the DSTs were no longer in use. After repeated contacts, 13 developers (representing 15 DSTs) agreed to participate in the study, resulting in an overall sample of 30 DSTs.

**Table 3:** IPDASi v3 Dimensions and Items.

Dimension	Item
**Information**	1. The decision support technology describes the health condition or problem (intervention, procedure or investigation) for which the index decision is required
Providing information about options in sufficient detail for making a specific decision	2. The decision support technology describes the decision that needs to be considered (the index decision)
	3. The decision support technology describes the options available for the index decision
	4. The decision support technology describes the natural course of the health condition or problem, if no action is taken.
	5. The decision support technology describes the positive features (benefits or advantages) of each option
	6. The decision aid describes negative features (harms, side effects or disadvantages) of each option.
	7. The decision support technology makes it possible to compare the positive and negative features of the available options.
	8. The decision support technology shows the negative and positive features of options with equal detail (for example using similar fonts, order, and display of statistical information).
**Probabilities**	1. The decision support technology provides information about outcome probabilities associated with the options (i.e. the likely consequences of decisions)
Presenting outcome probabilities	2. The decision support technology specifies the defined group (reference class) of patients for which the outcome probabilities apply.
	3. The decision support technology specifies the event rates for the outcome probabilities (in natural frequencies).
	4. The decision support technology specifies the time period over which the outcome probabilities apply.
	5. The decision support technology allows the user to compare outcome probabilities across options using the same denominator and time period.
	6. The decision support technology provides information about the levels of uncertainty around event or outcome probabilities (e.g. by giving a range or by using phrases such as “our best estimate is…”)
	7. The decision support technology provides more than one way of viewing the probabilities (e.g. words, numbers, and diagrams).
	8. The decision support technology provides balanced information about event or outcome probabilities to limit framing biases.
**Values**	1. The decision support technology describes the features of options to help patients imagine what it is like to experience the physical effects.
Clarifying and expressing values	2. The decision support technology describes the features of options to help patients imagine what it is like to experience the psychological effects.
	3. The decision support technology describes the features of options to help patients imagine what it is like to experience the social effects.
	4. The decision support technology asks patients to think about which positive and negative features of the options matter most to them.
**Decision Guidance**	1. The decision support technology provides a step-by-step way to make a decision.
Structured guidance in deliberation and communication	2. The decision support technology includes tools like worksheets or lists of questions to use when discussing options with a practitioner.
**Development**	1. The development process included finding out what clients or patients need to prepare them to discuss a specific decision
Using a systematic development process	2. The development process included finding out what health professionals need to prepare them to discuss a specific decision with patients
	3. The development process included expert review by clients/patients not involved in producing the decision support technology
	4. The development process included expert review by health professionals not involved in producing the decision aid.
	5. The decision support technology was field tested with patients who were facing the decision.
	6. The decision support technology was field tested with practitioners who counsel patients who face the decision.
**Evidence**	1. The decision support technology (or associated documentation) provides citations to the studies selected.
Using evidence	2. The decision support technology (or associated documentation) describes how research evidence was selected or synthesized.
	3. The decision support technology (or associated documentation) provides a production or publication date.
	4. The decision support technology (or associated documentation) provides information about the proposed update policy.
	5. The decision support technology (or associated documentation) describes the quality of the research evidence used.
**Disclosure**	1. The decision support technology (or associated technical documentation) provides information about the funding used for development.
Disclosure and transparency	2. The decision support technology includes author/developer credentials or qualifications.
**Plain Language**	1. The decision support technology (or associated documentation) reports readability levels (using one or more of the available scales).
Using plain language	
**DST Evaluation**	1. There is evidence that the decision support technology improves the match between the features that matter most to the informed patient and the option that is chosen
	2. There is evidence that the patient decision support technology helps patients improve their knowledge about options' features
**Test** (for DSTs that are directed at investigations or screening tests)	1. The decision support technology describes what the test is designed to measure.
	2. The decision support technology includes information about the chances of having a true positive test result.
	3. The decision support technology includes information about the chances of having a true negative test result.
	4. The decision support technology includes information about the chances of having a false positive test result.
	5. The decision support technology includes information about the chances of having a false negative test result.
	6. If the test detects the condition or problem, the decision support technology describes the next steps typically taken.
	7. The decision support technology describes the next steps if the condition or problem is not detected.
	8. The decision support technology describes the chances that the disease is detected with and without the use of the test.
	9. The decision support technology has information about the consequences of detecting the condition or disease that would never have caused problems if screening had not been done (lead time bias).

The time taken to assess a DST varies considerably, dependent on its complexity. A simple DST comprising a leaflet could be completed in two hours but assessing multimedia web-based DST required at least 8 hours. A weighted overall score (scaled from 0 to 100) for each DST is shown, averaged over two raters, and then adjusted for the pair of raters. Adjusted IPDASi scores ranged widely from 33 to 82 (Table 2). The intraclass correlation for the weighted overall score was 0.80. Correlations of dimension scores with the weighted overall score were all positive (0.31 to 0.68). Cronbach's alpha values for the 8 raters ranged from 0.72 to 0.93. Cronbach's alphas based on the means in the 9 dimensions ranged from 0.50 to 0.81, indicating that the dimensions, although relatively well correlated, measure different aspects of DST quality. Calculations of the standard deviation (SD) presenting imprecision using a Bayesian model based on the existing eight raters, and projected for different number of known (one of the existing eight raters used) and unknown raters, for whom we have no information about their scoring tendencies, resulted in the following estimates: two known raters, 6.6; one known rater, 9.4; two unknown raters, 9.3; one unknown rater, 13.1. Qualitative comments were received on some items, requesting clarifications. This was achieved by adding examples and more descriptive elements to the anchor statements.

### Stage 4 Agreement on IPDASi short form

The mean unweighted score for the short-form 16 item IPDASi was 56.1, similar to 56.3 for all items. The correlation of the unweighted IPDASi-SF to the overall mean weighted score (IPDASi score in Table 2) is 0.87 (CI 0.79–0.92). The ranking of the DSTs according to the SF version are very similar, with adjusted scores ranging from 34.5 to 83.1. DST number 32 still ranks highest, but the order shifts at the lower end of the scale. However, the aim of the IPDASi-SF was not to rank DSTs in order of quality but to determine whether or not a limited set of IPDASi items may be useful in determining minimal levels of quality.

## Discussion

### Principal Findings

This work demonstrates that IPDASi has the potential to assess the quality of DSTs. The four stage process revealed the need to make significant changes in the IPDAS checklist and modifications to the set of assumptions so that a measurement tool could be applied across the range of all possible DSTs. Having undertaken this work, we also suggest that IPDASi could provide formative feedback about dimensions in which DST developers could make improvements to subsequent versions. A short-form may also support the development of rapidly applicable quality standards. In addition, the study demonstrated the high correlation between IPDASi and IPDASi-SF, demonstrating support for the instrument's ability to provide correspondence between scores that indicate high quality at detailed dimension assessment and a version with focus on fewer items.

The study also displayed the levels of measurement imprecision when two raters assess each tool, and points to the need to ensure rater calibration and training in the use of IPDASi prior to assessment. We propose that IPDASi ratings should therefore be undertaken by raters who are familiar with DST development and use and who have undergone calibration training.

### Strengths and weaknesses

The instrument design is based on prior international consensus which provided a framework in which to assess DST quality, and in addition, a set of criterion-based ‘items’ for a new instrument. Secondly, the work was planned by researchers who followed a detailed protocol and met regularly. Thirdly, a staged approach was used, adopting the principles of instrument development [16]. Limitations of the study included the limited size of the sample and our focus on only DSTs developed in English, a constraint imposed by resource availability. There are also further opportunities to examine the validity of IPDASi, for example by examining whether low IPDASi scores for the ‘probability information’ dimension are associated with low patient knowledge about probabilities, when measured in controlled trials. Additionally, the raters used in the second and third stages were all researchers in the DST field and had some content expertise, so it is likely that raters with more diverse backgrounds may not perform as well. There was no opportunity in this study to provide intensive group training to all raters to ensure tight calibration and standardisation of item interpretation. To mitigate against this weakness, a detailed online manual that provided details about scale anchor definitions was available. Nonetheless, the results indicate that there is room to improve inter-rater reliability.

### Results in context

Two other studies have used the IPDAS checklist. Coulter et al undertook a detailed assessment of 40 information materials to support people in making decisions about their health and health care [17]. They found that the overall quality of information was poor and no systematic processes were adopted to give attention to presentational issues, such as readability or to ensure the validity of evidence. O'Connor et al used the checklist to assess the registered trials and found that several IPDAS process measures had not been used [13]. Williams used IPDASi v2 to assess DSTs for genetic testing for breast cancer [18]. We are not aware of any other work that has developed a quantitative measure of DST quality.

### Implications

IPDASi, and IPDASi-SF, will be available as a quality assessment method to developers, researchers and purchasers, and given a recognised need to set standards and achieve benchmarks, will be subject to further development. The existing IPDASi provides an assessment of the quality of a DST's components, and in the absence of any other method, will be used as a tool to provide formative advice to DSTs developers and as a summative assessment for those who want to compare their tools against existing benchmarks (http://www.ipdasi.org). In due course, data from these assessments might form a platform for potential certification but questions remain. There is for instance only one dimension on evaluation outcomes. The items in this dimension cannot be scored unless the developers have actually conducted an evaluation. It is likely that developers may assert that not all DSTs require evaluation, provided they meet other requirements. However, we contend that research in this field is at an early stage. There is no agreement as yet on the essential ‘active’ components of DSTs [19]; moreover the theoretical underpinning for both their mode of action, measurement models and implementation strategies needs strengthening [20], [21]. Further work is needed to assess which DSTs designs are superior to one another. Prospective studies that compare theoretically derived DSTs components and deliberation tools are required to help explore these areas.

The IPDAS collaboration and the resulting instruments (IPDASi and IPDASi-SF) need to meet the following challenges: How can new dimensions and items be considered? How are valid ‘option menus’ in DSTs derived and agreed when there are complex debates about equity, economics and evidence? Should there be items that assess the use of theory in the development of these methods, given that these are examples of ‘complex interventions’ and deserve attention to frameworks of design and mode of action [22]. These challenges provide an agenda for future research.

### What this paper adds

#### What is already known on this subject

Interest in decision support technologies is rapidly increasing and they are being accessed by ever larger number of patients, especially in the United States.

A quality *checklist* for decision support technologies has been published by the International Patient Decision Aid Standards Collaboration.

The checklist was not designed to provide precise, quantitative assessments about the quality these interventions.

#### What this study adds

Describes the development of an instrument which can assess the quality of decision support technologies, thereby enabling formative and summative feedback to developers and purchasers.
